# A Hierarchy of Functional States in Working Memory

**DOI:** 10.1523/JNEUROSCI.3104-20.2021

**Published:** 2021-05-19

**Authors:** Paul S. Muhle-Karbe, Nicholas E. Myers, Mark G. Stokes

**Affiliations:** ^1^Department of Experimental Psychology, University of Oxford, Oxford OX2 6GG, United Kingdom; ^2^Oxford Centre for Human Brain Activity, University of Oxford, Oxford OX2 6GG, United Kingdom; ^3^Wellcome Centre for Integrative Neuroimaging, University of Oxford, Oxford OX2 6GG, United Kingdom

**Keywords:** cognitive control, decision-making, decoding, EEG, representational states, working memory

## Abstract

Extensive research has examined how information is maintained in working memory (WM), but it remains unknown how WM is used to guide behavior. We addressed this question by combining human electrophysiology (50 subjects, male and female) with pattern analyses, cognitive modeling, and a task requiring the prolonged maintenance of two WM items and priority shifts between them. This enabled us to discern neural states coding for memories that were selected to guide the next decision from states coding for concurrently held memories that were maintained for later use, and to examine how these states contribute to WM-based decisions. Selected memories were encoded in a functionally active state. This state was reflected in spontaneous brain activity during the delay period, closely tracked moment-to-moment fluctuations in the quality of evidence integration, and also predicted when memories would interfere with each other. In contrast, concurrently held memories were encoded in a functionally latent state. This state was reflected only in stimulus-evoked brain activity, tracked memory precision at longer timescales, but did not engage with ongoing decision dynamics. Intriguingly, the two functional states were highly flexible, as priority could be dynamically shifted back and forth between memories without degrading their precision. These results delineate a hierarchy of functional states, whereby latent memories supporting general maintenance are transformed into active decision circuits to guide flexible behavior.

**SIGNIFICANCE STATEMENT** Working memory enables maintenance of information that is no longer available in the environment. Abundant neuroscientific work has examined where in the brain working memories are stored, but it remains unknown how they are represented and used to guide behavior. Our study shows that working memories are represented in qualitatively different formats, depending on behavioral priorities. Memories that are selected for guiding behavior are encoded in an active state that transforms sensory input into decision variables, whereas other concurrently held memories are encoded in a latent state that supports precise maintenance without affecting ongoing cognition. These results dissociate mechanisms supporting memory storage and usage, and open the door to reveal not only where memories are stored but also how.

## Introduction

Working memory (WM) refers to the ability to maintain and manipulate information that is no longer available in the environment. It provides a flexible mental workspace that scaffolds many higher cognitive functions such as planning, reasoning, or cognitive control (Oberauer, 2009). Therefore, a long-standing theme in cognitive neuroscience has been to delineate neural mechanisms that underpin WM. This research has made great progress in revealing how the brain maintains information across delay periods, for example, via persistent activity (Wimmer et al., 2014), neuronal oscillations (De Vries et al., 2017), or activity-silent brain states (Rose et al., 2016; Stokes, 2015). At the same time, however, this work has so far neglected a key aspect of WM, namely, how its contents are used to guide flexible behavior.

Previous studies have documented dissociations between the maintenance and use of WM items (Muhle-Karbe et al., 2017). Patients with frontal lobe damage sometimes exhibit a phenomenon termed goal neglect, wherein instructed task components are disregarded during performance, although they have been understood and remembered (Duncan et al., 1996). Studies in healthy humans furthermore suggest that WM items that will guide the next decision can bias perception toward memory-matching stimuli, while other concurrently held items do not affect ongoing cognition (Olivers et al., 2011). Likewise, in neuroimaging studies, immediately task-relevant WM items can be robustly decoded from brain activity patterns, while concurrently held items typically exhibit little or no decodability (Lewis-Peacock et al., 2015). Such findings are commonly thought to reflect the effects of attention, which may prioritize a single item in WM by amplifying the corresponding neural patterns (Chun, 2011; LaRocque et al., 2014). This view echoes the tenet of theories conceiving WM storage as the distribution of a limited cognitive resource (Zhang and Luck, 2008; Ma et al., 2014). From this perspective, selecting an item for guiding behavior focuses resources and boosts the strength of the selected item, while degrading other concurrently held items.

An alternative proposal states that the use of WM does not rely on attentional selection alone, but also entails a reconfiguration of representational formats from a purely mnemonic state into an action-ready state that is tuned for efficient task-dependent decision-making (Muhle-Karbe et al., 2017; Myers et al., 2017). From this perspective, WM representations should exhibit qualitatively different functional attributes depending on their momentary task relevance. Immediately task-relevant items should be encoded in a functionally active state that is optimized for task-dependent readout. By contrast, items that are only prospectively task relevant should be held in a functionally latent state that supports maintenance but does not modulate cognition until behavioral priorities change. This view predicts that active and latent states both support WM-guided behavior, but at different timescales with latent states supporting maintenance over the intermediate term and active states biasing moment-to-moment processing to guide memory-based decisions.

We tested this view using electroencephalography (EEG) and a novel task that allowed us to compare these two putative functional states. Participants maintained two WM items for an extended time period, though at any given moment only one item was immediately task-relevant (cued item), while the other item was maintained for later use (uncued item). We leveraged a combination of multivariate decoding analyses and cognitive modeling to recover representations of cued and uncued WM items and link variance in decoding strength with variance in performance. Consistent with our proposal, we find that cued and uncued items are encoded in largely dissociable neural patterns. Patterns coding for uncued items reflect a functionally latent state that tracks memory precision at longer timescales but is unrelated to trial-wise performance variability. By contrast, the unique pattern component coding for cued items reflects a functionally active state that closely tracks trial-wise variance in the quality of evidence integration for WM-based decisions. Together, these findings suggest a hierarchy of functional states in WM, wherein functionally latent memories supporting general maintenance are transformed into functionally active decision circuits to guide flexible behavior.

## Materials and Methods

### 

#### 

##### Participants.

Fifty healthy adults participated in two experiments. Twenty participants took part in experiment 1 (mean age = 28.1 years; age range = 18–37 years; 10 females, 1 left handed), and 30 participants took part in experiment 2 (mean age = 26.8 years; age range = 19–41 years; 14 females; 2 left handed). Seven participants took part in both experiments. Sample sizes were similar to previous studies from our group (Wolff et al., 2017), but they were not determined based on a formal power analysis. All subjects reported normal or corrected-to-normal vision and received a monetary compensation for participation. The study was approved by, and conducted in accordance with, the guidelines of the Central University Research Ethics Committee of the University of Oxford. As we were interested in potential null effects (e.g., absence of trial-wise links between the decoding strength of uncued items and performance), we decided to pool the data across both experiments for several analyses to maximize statistical power and our ability to detect even subtle effects. In those analyses, we averaged the data from subjects who participated in both experiments across the two sessions, resulting in 43 unique datasets for pooled analyses.

##### Apparatus.

Stimulus presentation was controlled in MATLAB using Psychtoolbox on a 22 inch monitor with a refresh rate of 100 Hz. Unless reported otherwise, stimuli were shown in white font on a gray background (50% contrast; RGB = 127, 127, 127) at a distance of ∼60 cm. Responses were given with the left and right index fingers on the “B” and “Y” buttons of a QWERTY keyboard. EEG data were collected using 61 channels that were distributed across the scalp according to the extended 10–20 positioning system. Data were collected at 1000 Hz using a NeuroScan SynAmps RT Amplifier and Curry 7 software. Impedances of all channels were kept to <5 kΩ. In both studies, eye movements were recorded via electro-oculography (EOG) using electrodes places above and below the left eye, to the left of the left eye, and to the right of the right eye. In study 2, we additionally recorded eye movements using a remote infrared eye-tracker (Eyelink 1000, SR Research) sampling both eyes at 1000 Hz. We also recorded activity in the first dorsal interosseus muscle of the left and right hand via electromyography (EMG) at 1000 Hz.

##### Experimental design and statistical analysis.

The experimental task required the prolonged maintenance of two items in WM and flexible priority shifts between those items to guide task-dependent decision-making (see Fig. 2, illustration). Overall, the task was broken into blocks each consisting of 16 trials. At the beginning of each block, participants were shown two orientated bars (length, 6° visual angle; width, 0.25° visual angle; presented at the same location in the center of the screen as subsequent stimuli) in blue (RGB = 25.5, 25.5, 204) and yellow (RGB = 204, 204, 25.5) color. These two bars served as memory items for the remainder of the block. For each participant, one color was associated with a high-pitch tone and the other color was associated with a low-pitch tone (mapping counterbalanced across participants). The two orientations were drawn randomly from a set of 16 possible orientations (spaced evenly at 11.25° intervals from 2.8125° to 171.5625°) with the sole constraints that the orientation of the two items could not be identical or exactly orthogonal. Participants could encode the two items for a duration of their choosing and initiated the block via button press. Within the block, each trial started with a presentation of an auditory cue (pure sinusoidal tones: low tone, 440 Hz; high tone, 880 Hz; duration: 100 ms including a 10 ms ramp-up and 10 ms ramp-down). The cue identity signaled which memory item should be used as a boundary for a forthcoming perceptual decision (cued item), while the other item was maintained merely for later use in the block (uncued item). The cue was followed by a 700 ms delay period within which a black fixation dot was presented centrally on the screen (diameter, 0.15°). Thereafter, a randomly oriented Gabor patch was presented (orientation drawn from a set of 16 possible orientations, spaced evenly at 11.25° intervals from 8.4375° to 177.1875°; patches: 6° diameter; 50% contrast; 1.75 cycles/°; random phase, Gaussian envelope with 1.2° SD). Participants were given a maximum time window of 4000 ms to classify the target via button press as being tilted clockwise or counterclockwise relative to the cued item. Targets were presented for 100 ms and replaced by a fixation dot for the remainder of the response period. In experiment 1, the target was presented centrally on the screen on all trials. In experiment 2, the target was presented laterally at a distance of 6° from the screen center. The side of target presentation (left vs right) alternated predictably across blocks. The change in stimulus position in experiment 2 was implemented to facilitate stable fixation during the target period and minimize a potential contamination of the EEG signal by eye movements toward the target (Mostert et al., 2018). A noise patch (Gaussian smoothed random white noise using a kernel with 0.13° SD, convolved with a Gaussian envelope with 1.2° SD) that matched the target stimulus in luminance, size, contrast, and eccentricity was presented on the side of the screen at which no target appeared (see Fig. 2*B*, illustration). Clockwise and counterclockwise decisions were indicated with the right and left index fingers, respectively. The response period was followed by a variable intertrial interval (400–900 ms; drawn from a truncated exponential distribution: mean, 550 ms) until the next trial started with the presentation of a cue. Importantly, participants received feedback about their performance only at the end of each block when their mean accuracy and response time from the preceding block was presented. Accordingly, they had to maintain precise representations of both memory items for the whole duration of the block, as they could not rely on trial-wise feedback to infer the orientation of an item if it was forgotten. Overall, participants completed 128 blocks, resulting in a total of 2048 trials and lasting ∼2 h.

##### Behavioral data analyses.

Performance accuracy and log-transformed reaction time (log-RT) was analyzed using general linear models (GLMs). Initially, we tested to what extent performance was affected by the angular distance between the orientation of the target and the orientation of the cued WM item. We next repeated the analysis, but this time testing for an effect of the distance between target orientation and the orientation of the uncued WM item. To visualize these results, we fit the proportion of clockwise choices with a binomial cumulative distribution function using a GLM implemented in R with the ggplot2 and the psyphy packages (Webster et al., 2019). We fit another GLM to test for the presence of performance costs as a result of priority shifts within WM by comparing accuracy and RT between trials that incurred a shift in priority (switch trials) and trials that did not (repetition trials). Finally, we tested the stability of performance within and across experimental blocks by predicting accuracy and log-RT based on a variable denoting the trial number within a block (1–16) and another variable denoting the block number within the experiment (1–128).

##### Preprocessing.

EEG data were initially rereferenced to the average of both mastoids. EEG, EOG, and EMG data were then downsampled to 250 Hz and bandpass filtered, using a high-pass filter of 0.1 Hz and a low-pass filter of 45 Hz. Because of recent evidence suggesting that excessive high-pass filtering can yield temporal displacement of decoded information (van Driel et al., 2019), we repeated all of our analyses with a more lenient high-pass filter of 0.01 Hz, which yielded nearly identical results. EEG channels with excessive noise were identified through visual inspection and replaced via interpolation using a weighted average of the surrounding electrodes. The continuous time series data were then divided into epochs, corresponding to the experimental trials starting 200 ms before to the onset of the cue and terminating 1800 ms after the onset of the target. Each trial was inspected visually for blinks, eye movements, and nonstereotyped artifacts. Trials were rejected if they contained any of those artifacts during the delay and/or target period. Stereotyped artifacts outside those periods were subsequently removed from the data via independent component analysis. Unless stated otherwise, the data were baseline corrected for the decoding analyses using the average signal from the time window of 200–50 ms before cue onset. Eye-tracking data were downsampled to 250 Hz, and we identified and interpolated blinks using spline interpolation and a time window of ±100 ms around the event (van Ede et al., 2019).

##### EEG decoding analyses.

We conducted a series of multivariate pattern analyses to characterize the neural representations that underpin performance in our task.

##### Time-resolved decoding.

**Figure 1.**
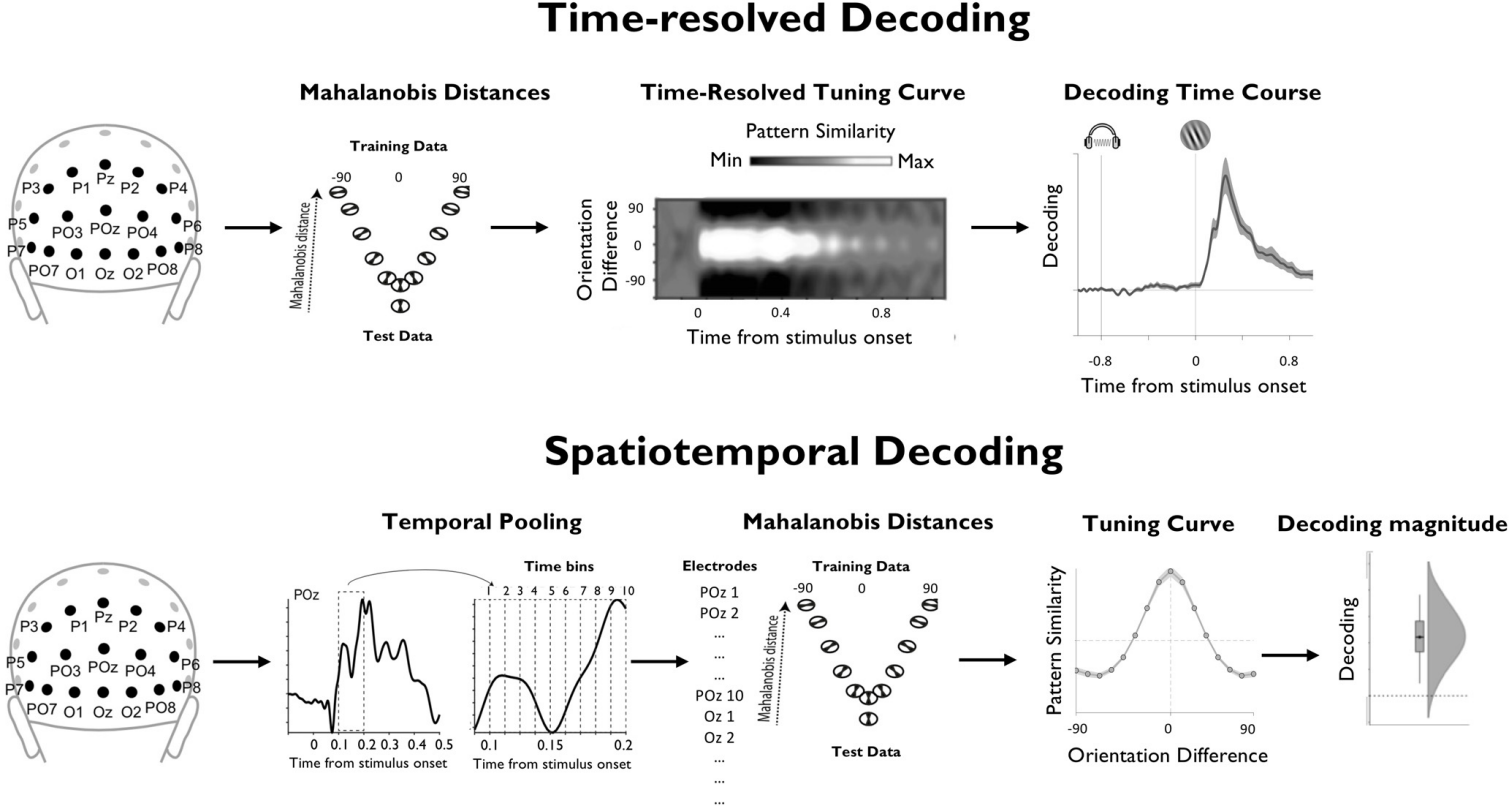
Illustration of time-resolved and spatiotemporal approaches that were used for the decoding of task variables. Top, Illustration of the time-resolved decoding approach. Here, for each training set and time point, Mahalanobis distances were computed between activation patterns of posterior sensors of the test data and the training data (binned based on their orientation relative to the test data). After repeating this procedure for every training set and time point, the resulting distances were sign reversed, so that positive scores reflect pattern similarity, and were centered around the mean voltage across sensors for each time point. This was used to reconstruct a time-resolved population tuning curve that expresses the extent to which pattern similarity reflects the similarity of the underlying angular orientation space across time. We estimated the height of this tuning curve for each time point by convolving it with a cosine function (for details, see Materials and Methods). The resulting vector served as an index of time-resolved decoding accuracy. Bottom, Illustration of the spatiotemporal decoding approach. Here, the data entering the decoders were pooled over multiple time points, so as to exploit not only information that is encoded in spatial activation patterns, but also information that is encoded in their temporal structure. We focused on the time window from 100 to 400 ms after target onset and treated individual trials as discrete events. Tuning curves were estimated based on mean-centered and sign-reversed Mahalanobis distances, as in the time-resolved approach, yielding a single index of decoding strength for each trial as final output.

In a first step, we conducted a time-resolved decoding analysis to reveal the time courses at which three variables of our task were explicitly encoded in EEG sensor activity, as follows: (1) the orientation of the target stimulus; (2) the orientation of the cued and uncued memory items; and (3) a decision variable that was calculated as the absolute distance between the orientation of the target and the orientation of the two memory items. To recover parametric information about these variables from EEG sensor activity with high temporal resolution, we computed Mahalanobis distances between the patterns of sensor activity that were evoked by different stimulus orientations and measured the extent to which these distances reflected the underlying circular orientation space (or linear decision variable space; Fig. 1). We used a leave-one-block-out cross-validation procedure with the data from each block serving as test data once and all of the remaining data serving as training data. Training data were subdivided into 16 bins, according to their orientation relative to the test data, and then averaged. Mahalanobis distances between the 16 average patterns in the training data and each test trial data were then computed using the noise covariance from training data. Noise covariance was calculated on the average residual data after subtracting orientation-specific mean activity from each trial with the corresponding orientation. Residuals were averaged over a time window from 0 to 1.8 s relative to the onset of the auditory cue (and therefore encompassed the cue, delay, target, and decision phases). The covariance was calculated over the trials-by-sensors matrix of average residuals, using a shrinkage estimator (Ledoit and Wolf, 2004). This procedure was repeated for every block and for every time point within the trial using 4 ms time bins. We normalized the resulting pattern distances by subtracting the mean across the 16 distances for each time point from the activity of each sensor. To simplify interpretation, we reversed their sign so that positive values reflected pattern similarity rather than distance. This procedure enabled us to compute an orientation tuning curve for each time point and trial, which expresses the extent to which pattern similarity decreases as a function of angular distance. To transform the 16-dimensional tuning curve into a 1-dimensional index of decoding accuracy, we computed the cosine vector mean of each tuning curve by rescaling the cosine of the center of each orientation bin to the range from −180 to 180 and multiplying it with the corresponding pattern similarities. The mean of the resulting 16 values served as an index of decoding accuracy with positive values reflecting the tuning of the EEG signal for stimulus orientation (Sprague et al., 2016; Wolff et al., 2017). Decoding values were smoothed with a Gaussian kernel (SD = 24 ms) for visualization and significance testing. We tested for significance using one-sample *t* tests against 0 at each time point, and corrected for multiple comparisons in time via cluster-based permutation testing using 10,000 permutations (Maris and Oostenveld, 2007). For the decoding of the memory items, cluster correction was applied for the whole trial. In contrast, for the decoding of the target and the decision variable, cluster correction was only applied for the target period (i.e., the 1800 ms after target onset), because these variables could by definition only be encoded after the onset of the target stimulus. In keeping with previous work from our group (Wolff et al., 2020a,b), decoding analyses were conducted only within posterior EEG sensors (P7, P5, P3, P1, P2, P4, P6, P8, PO7, PO5, PO3, POz, PO4, PO8, O1, Oz, O_2_). We selected these channels to be consistent with previous studies and because orientation signals are typically expressed most strongly in posterior regions (Cichy et al., 2015; Myers et al., 2015; Kok et al., 2017). Please note, however, that none of the reported results depend on this channel subselection (i.e., all reported effects remain significant when using all 61 channels). Moreover, please note that, in experiment 2, decoding analyses were conducted separately for blocks with target presentation on the left and right side, and decoding accuracies were subsequently averaged across sides. The decision variable (absolute difference between memory item and target) followed a discrete uniform distribution between 5.625° and 84.375°, unlike the other variables (memory items and target stimuli), which had a circular distribution. Our decoding approach therefore needed to be adjusted for decoding of the decision variable. Instead of reducing the dimensionality of the tuning curve using a cosine vector mean, we calculated the linear slope of the tuning curve. Notably, previous studies have reported that decoded memory signals can be contaminated by stimulus-specific eye movements (Mostert et al., 2018). We therefore repeated the aforementioned decoding analyses with data from the binocular EOG channels (experiment 1) or eye-tracker channels (experiment 2) to reveal whether the same task variables were encoded in participants' eye positions. We also regressed the eye channel-based decoding time series against the EEG-based decoding time series to reveal the extent to which decoded EEG signals were linked with potential concomitant oculomotor signals.

##### Spatiotemporal decoding from stimulus-evoked EEG signals.

In a second step, we used a complementary analysis approach to decode the same task variables from longer time windows of stimulus-evoked brain activity. This approach exploits the dynamic temporal structure of event-related potentials by pooling multivariate information in time, thus capturing not only information encoded in spatial activation patterns, but also information that is encoded in the temporal unfolding of these spatial patterns. A previous study from our group has shown that such pooling over adjacent time points within trials can increase decoding sensitivity at the expense of temporal precision (Wolff et al., 2020a). Consequently, the two decoding approaches capture complementary aspects of neural representations and should be considered mutually informative. In keeping with the previous study, we combined EEG data from the 17 posterior channels within a time window of 100–400 ms after target onset, thus treating single trials as discrete events. The time window was chosen because stimulus-evoked WM signals are largely confined to this period (Wolff et al., 2017, 2020a,b). The mean activity at each sensor and time point was removed to normalize voltage fluctuations and isolate dynamic, stimulus-evoked brain states from brain activity that is stable across the chosen time period. To avoid having more data features (number of sensors × number of time points in the training data) than data samples (number of trials in the training set), we also downsampled the signal at each sensor by a factor of 10 (i.e., from 250 to 25 Hz). Following this preparation, we performed decoding analyses via Mahalanobis distances using the same procedure as detailed above. The spatiotemporal approach was used to decode the same task variables (target stimulus, memory items, decision variable), yielding a single vector of decoding accuracies for each subject and task variable. We evaluated the significance of results via one-sample *t* test against 0 (one-tailed, testing for above-chance decoding).

##### Regression analyses.

**Figure 2.**
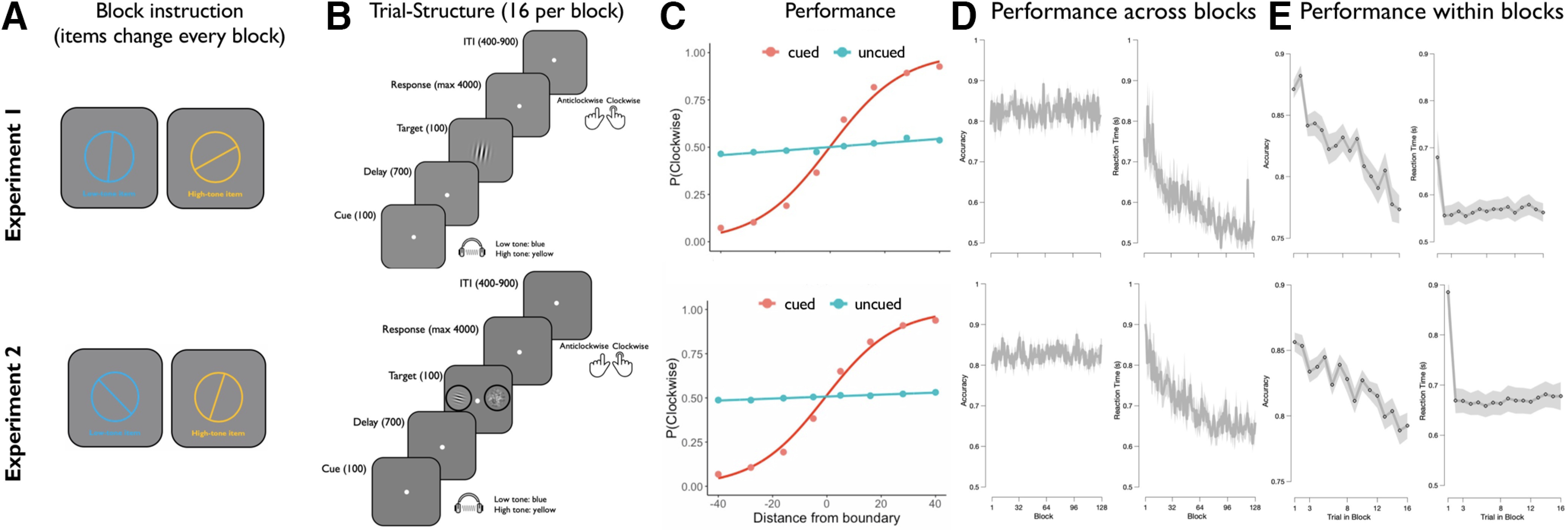
Illustration of the task design and behavioral results of experiment 1 (top) and experiment 2 (bottom). ***A***, At the beginning of each block, two randomly oriented bars were shown successively in blue and yellow. These two items served as memory items for the remainder of the block and were associated with a high-pitch tone and a low-pitch tone, respectively (mapping counterbalanced across subjects). Please note that the items were successively presented at the center of the screen. ***B***, During the block, each trial started with the presentation of an auditory cue that signalled which item should be used as the decision criterion on the current trial (cued item), while the other item had to be maintained for later use in the block (uncued item). After a brief delay, a randomly oriented Gabor patch was presented as the target, and participants were required to indicate whether the target was oriented clockwise or counterclockwise relative to the cued item. In experiment 1, the target was shown centrally on the screen. In experiment 2, the target was shown peripherally on the left or right side of the fixation dot, while a noise patch was presented on the other side. The noise patch matched the target in luminance, size, contrast, and eccentricity. Note that the location of the target was indicated to participants at the beginning of each block. ***C***, Probability of clockwise responses as a function of the angular difference between target stimulus and the currently cued item (shown in red) and from the currently uncued item (shown in blue). Data are shown in dots, and the lines represent a fitted binomial cumulative distribution function. Responses were strongly modulated by the angular distance between the orientation of the target and the orientation of the cued item, whereas the distance between the target and uncued item had only minimal impact on performance (for details, see Materials and Methods, and Results). ***D***, ***E***, Performance stability across and within experimental blocks. Overall, participants completed 128 blocks, each containing 16 trials. Plots on the left display performance as a function of block number. While accuracy was stable across blocks, RT continuously declined with increasing block numbers. Conversely, within blocks, accuracy continuously declined, whereas RT was stable, with the exception of the first trial, which exhibited longer RT than subsequent trials. Shadings indicate SEMs.

We conducted a series of regression analyses to establish the behavioral relevance of the decoded memory signals and test our hypothesis that cued and uncued memory items are encoded in qualitatively different functional states. In a first step, we regressed log-RT and accuracy against the trial-wise decoding strength of cued and uncued memory items. That is, for each trial and item, we calculated the decoding strength using the spatiotemporal approach described above, and normalized these scores by subtracting the block average to isolate across-trial fluctuations from more sustained changes in decoding. We then used these normalized scores to predict trial-wise variance in performance with general linear models serving to predict log-RT and logistic regression models serving to predict accuracy. We also included regressors for the block number and the trial within the block to account for general changes in performance across and within blocks (see Results; Fig. 2*D*,*E*). As we were interested in potential null effects (i.e., absence of links between trial-wise variance in performance and trial-wise variance in the decoding strength of uncued items), we analyzed regression weights through a combination of frequentist and Bayesian statistics. Initially, we tested whether regression weights were significantly different from 0 using one-sample *t* tests and also calculated Bayes factors (BFs) from Bayesian one-sample *t* tests to quantify the evidence in favor of the null hypothesis and the alternative hypothesis. Based on our hypotheses, we used one-tailed tests in the direction of facilitation (i.e., positive regression weights for accuracy and negative weights for log-RT) for the cued item and in the direction of interference (i.e., negative weights for accuracy and positive weights for log-RT) for the uncued item. These analyses were implemented within JASP software using default priors for the Bayesian analyses (Keysers et al., 2020). We also compared regression weights between cued and uncued items using paired *t* tests (one-tailed, testing for greater facilitation with cued items) and calculated Bayes factors resulting from a Bayesian paired-samples *t* test.

In a second step, we regressed log-RT and accuracy against the average block-wise decoding strength of each memory item. That is, we predicted performance on each trial (test trial) based on the average decoding strength across all remaining trials within the same block on which the respective memory item was cued or based on the average decoding strength across all trials on which the respective memory item was uncued. Analogously to the trial-wise analyses, we calculated frequentist and Bayesian statistics to analyze the resulting regression weights.

Finally, we repeated the trial-wise analysis with the output from a cross-item decoder that was trained with data sorted by the cued item and tested with data sorted by the uncued item. This analysis aimed to reveal whether interference from uncued items scales with the extent to which these items are encoded in neural patterns that resemble their functionally active state. Initially, regression weights were tested against chance level using one-sample *t* tests (one-tailed testing for above-chance decoding) and compared with the regular decoder of the uncued item using paired samples *t* tests and Bayes factors (two-tailed, based on the lack of a priori predictions). Thereafter, the regression weights of the cross-item decoder were tested against 0 using one-sample *t* tests and Bayes factors (one-tailed testing for interference effects). We repeated this analysis separately for trials that demanded a priority shift between WM items (switch trials), relative to the previous trial, and trials that did not (repetition trials). Regression weights were compared between trial types via paired-samples *t* tests (one-tailed, testing for greater interference effects on switch trials). We also conducted a control analysis to match the cross-item decoder and the regular decoder of the uncued item in terms of mean and SD. To this end, we first mean centered the two vectors that denoted trial-wise scores of each decoder. Mean-centered vectors were then scaled by the SD of the cross-item decoding vector. We then calculated the SD of the noise that was required to match the scaled cross-item decoder with the scaled regular decoder of the uncued item and added random noise with the estimated properties to the output of the cross-item decoder. The noise-matched decoder was then used to predict performance using the method described above for trial-wise regression analyses. This procedure was repeated with 1000 noise injections and regression weights were averaged across all iterations.

##### Drift diffusion modeling.

In the next set of analyses, we aimed to characterize functionally active WM states in more detail by delineating how they influenced WM-guided decisions in our task. To this end, we fit a set of drift diffusion models (DDMs) to our behavioral data and regressed variance in model parameters against variance in decoding strength. DDMs characterize decisions as the accumulation of noisy evidence between two competing options, whereby one option is chosen once a sufficient amount of evidence has been accumulated (Ratcliff and McKoon, 2008; Shepherdson et al., 2018). DDMs decompose performance in two-alternative choice tasks into latent decision parameters based on RT distributions and choice probabilities. The most parsimonious version of the DDM has the following three parameters: drift rate, non-decision time, and decision threshold. First, the drift rate reflects the quality or strength of decision-relevant information and scales negatively with categorization difficulty (i.e., lower drift rate with more difficult discriminations). Second, the non-decision time is thought to reflect the time needed for processes that are not directly related to evidence accumulation such as the encoding of a stimulus or the execution of a response. Finally, the decision threshold (or boundary separation) reflects the amount of evidence that is needed to commit to a behavioral choice. This parameter is thought to be under strategic control and to regulate speed–accuracy tradeoffs (e.g., higher thresholds will result in slower but more accurate responses).

After observing that trial-wise variance in the decoding strength of cued, but not uncued, memory items tracked variance in task performance (see Results), the central aim of this analysis was to identify which decision parameter could account for this benefit. This enabled us to compare the following two hypotheses that have previously been proposed in the literature: (1) the matched filter hypothesis; and (2) the retrieval head-start hypothesis. Under the matched filter hypothesis, variance in decoding strength should track the ease with which sensory input is interpreted and transformed into a decision variable. This would be the case, for example, if the same neuronal population that encodes cued items also processes incoming stimuli and computes a signal reflecting their match/mismatch (Sugase-Miyamoto et al., 2008; Hayden and Gallant, 2013). This view predicts that variance in decoding strength across trials should be positively associated with variance in drift rate (see Fig. 8*A*). Alternatively, under the retrieval head-start hypothesis, prioritization of WM items primarily affects their accessibility for decision-making, thus reducing the latency at which evidence accumulation can start. This view predicts that variance in decoding strength across trials should scale negatively with variance in non-decision time (see Fig. 8*B*).

To test these hypotheses, we conducted a series of analyses using the HDDM python toolbox in version 0.6 (Wiecki et al., 2013). HDDM implements a hierarchical Bayesian version of the DDM, wherein model fits for individual participants are constrained by the group distribution. It applies Markov Chain Monte Carlo sampling methods to estimate posterior distributions over DDM parameters. Each parameter was modeled to be normally distributed and centered around the group mean. Moreover, the prior distribution for each parameter was informed by a collection of 23 empirical studies documenting decision parameters with optimal fit across a range of decision paradigms (Wiecki et al., 2013). Five thousand samples were taken from each distribution, from which the first 1000 were discarded as burn-in. This was done because the initial samples are likely to be unreliable because of the random selection of a starting point. To establish links between trial-wise fluctuations of the decision parameters and trial-wise fluctuations in the decoding strength of the cued memory item, we fit a set of linear regression models using the patsy library. Separate analyses were run for drift rate and non-decision time. We evaluated results statistically via the posterior distribution of the estimated regression weights. Effects were considered significant if at least 95% of the posterior probability mass was above or below 0, and in the predicted direction (positive for drift rate and negative for non-decision time; see explanation above).

##### Effects of priority shifts on memory precision.

In the final set of analyses, we aimed to characterize the format of functionally latent WM states in more detail by testing whether shifting priority away from a WM item would degrade its precision, relative to maintaining the same item in a prioritized state. Such shift-dependent degradation is predicted by resource theories of WM, which assume that items receive fewer mnemonic resources when they are maintained in a nonprioritized state. This prediction contrasts with state-based theories, which assume that prioritized and nonprioritized WM items are represented with different functional attributes but similar precision. Hence, by probing the potential effects of priority shifts on memory precision, we could compare different accounts of WM prioritization that are based on the distribution of mnemonic resources or the reconfiguration of functional states.

To examine this question, we conducted a series of behavioral and EEG analyses. Initially, we performed another regression analysis to test whether the number of priority shifts that had preceded a given trial within a block would predict a decline in performance above and beyond a purely time-dependent decline (as measured by the trial number within the blocks). We predicted accuracy and log-RT using a GLM that contained predictor variables for the trial number within the current block (block trial) and the number of cue switches preceding the current trial within the block (cue sequence; see Fig. 9*A*). The block trial variable indexes time-dependent degradation, whereas the cue sequence variable indexes shift-dependent degradation. As for the previous analyses, we evaluated regression weights of each predictor variable using one-sample *t* tests (one-tailed in the direction of degradation) and Bayes factors from Bayesian one-sample *t* tests.

In a second step, we tested whether shifting priority away from an item would increase categorical biases, whereby items are encoded with respect to their closest cardinal boundary. We therefore calculated a variable reflecting the angular distance between the cued WM item on each trial and the closest cardinal axis (vertical or horizontal; see Fig. 9*B*). This variable was used as a predictor for accuracy and log-RT, separately for each cue sequence. Regression weights were again evaluated using one-sample *t* test (one-tailed in the direction of categorical biases) and Bayes factors, and compared between cue sequences using paired-samples *t* tests and Bayes factors. Paired-samples *t* tests were one-tailed testing for shift-dependent degradation (i.e., larger effects of cardinal distance with later cue sequences).

Finally, we also tested effects of priority shifts on WM representations using EEG-based decoding strength as an index of WM precision. We therefore divided decoder outputs based on the first four cue sequences within each block (see Fig. 9*A*). To test for differences in decoding time course, we compared the time-resolved decoding strength between the four cue sequences using the cluster correction described above. We also compared the spatiotemporal decoding strength (see description above) between the first four cue sequences using paired-samples *t* test and Bayes factors (one-tailed, testing for shift-dependent degradation). Last, we also conducted regression analyses, predicting log-RT and accuracy from spatiotemporal decoding strength, separately for each cue sequence. As for the previous analyses, regression weights were tested against chance using one-sample *t* tests and Bayes factors, and compared using paired-samples *t* tests and Bayes factors (one-tailed, testing for decreases in performance prediction with larger cue sequences).

##### Data availability.

The data, task, and analysis scripts from this study will be made publicly available at https://osf.io/v28fm/?view_only=b8ebb32d8242455c9f9c37ccaceab76b.

## Results

### Behavior

Participants were able to perform the task well above chance (experiment 1: accuracy = 79.5%; reaction time = 573 ms; experiment 2: accuracy = 80.1%; reaction time = 679 ms). As shown in Figure 2*C*, performance was strongly modulated by the angular distance between the target orientation and the orientation of the cued WM item. RT decreased with greater distances (experiment 1: *t*_(19)_ = 10.078; *p* < 0.001; Cohen's *d* = 2.253; experiment 2: *t*_(29)_ = 10.462; *p* < 0.001; *d* = 1.910), while accuracy increased (experiment 1: *t*_(19)_ = 12.677; *p* < 0.001; *d* = 2.835; experiment 2: *t*_(29)_ = 18.426; *p* < 0.001; *d* = 3.364). By contrast, the angular distance between the target and the uncued WM item had only marginal impact on RT (experiment 1: *t*_(19)_ = 2.49; *p* = 0.069; *d* = 0.441; experiment 2: *t*_(29)_ = 2.125; *p* = 0.103; *d* = 0.325), and a modest effect on accuracy (experiment 1: *t*_(19)_ = 2.751; *p* = 0.013; *d* = 0.615; experiment 2: *t*_(29)_ = 0.205; *p* = 0.839; *d* = 0.037). In addition, we observed a reliable effect of priority shifts, whereby performance was slower (experiment 1: *t*_(19)_ = 7.097; *p* < 0.001; *d* = 1.587; experiment 2: *t*_(29)_ = 12.864; *p* < 0.001; *d* = 2.349) and more error prone (experiment 1: *t*_(19)_ = −2.704, *p* = 0.014, *d* = −0.605; experiment 2: *t*_(29)_ = −2.711, *p* = 0.011, *d* = −0.495) on trials that incurred a shift in priority between the two WM items (experiment 1: RT = 631 ms; accuracy = 78.9%; experiment 2: RT = 772 ms; accuracy = 79.5%), relative to the previous trial, than on trials in which the item priority repeated (experiment 1: RT = 551 ms; accuracy = 79.7%; experiment 2: RT = 646 ms; accuracy = 80.3%). We also found that accuracy was stable across experimental blocks (experiment 1: *t* = 1.214; *p* = 0.240; *d* = 0.271; experiment 2: *t*_(29)_ = 0.080; *p* = 0.937; *d* = 0.015), but declined continuously across trials within blocks (experiment 1: *t*_(19)_ = −13.841; *p* < 0.001; *d* = 3.095; experiment 2: *t*_(29)_ = −6.242; *p* < 0.001; *d* = −1.140). Conversely, RT decreased with increasing block numbers (experiment 1: *t* = −7.227; *p* < 0.001; *d* = −1.616; experiment 2: *t* = −7.129; *p* < 0.001; *d* = 1.302), but was stable within blocks except for the first block trial (experiment 1: *t*_(19)_ = −0.432; *p* = 0.671; *d* = −0.097; experiment 2: *t*_(29)_ = −2.528; *p* = 0.017; *d* = −0.462; Fig. 2*D*,*E*)

### Time-resolved decoding of task variables

We conducted a series of multivariate pattern analyses to characterize the neural representations that underpin performance in our task. In a first step, we conducted a time-resolved decoding analysis to reveal the time courses within which the following three task variables were explicitly encoded in EEG sensor activity: (1) the orientation of the target stimulus; (2) the orientation of the memory item, separately for cued and uncued conditions; and (3) a decision variable that was calculated as the absolute distance between the orientation of the target and the orientation of the WM item, again separately for cued and uncued conditions. To recover information about these variables from EEG sensor activity with high temporal resolution, we computed Mahalanobis distances between the patterns of sensor activity that were evoked by different stimulus orientations and measured the extent to which these distances reflected the underlying circular orientation space or linear decision variable space (Fig. 1, illustration; for details, see Materials and Methods).

**Figure 3.**
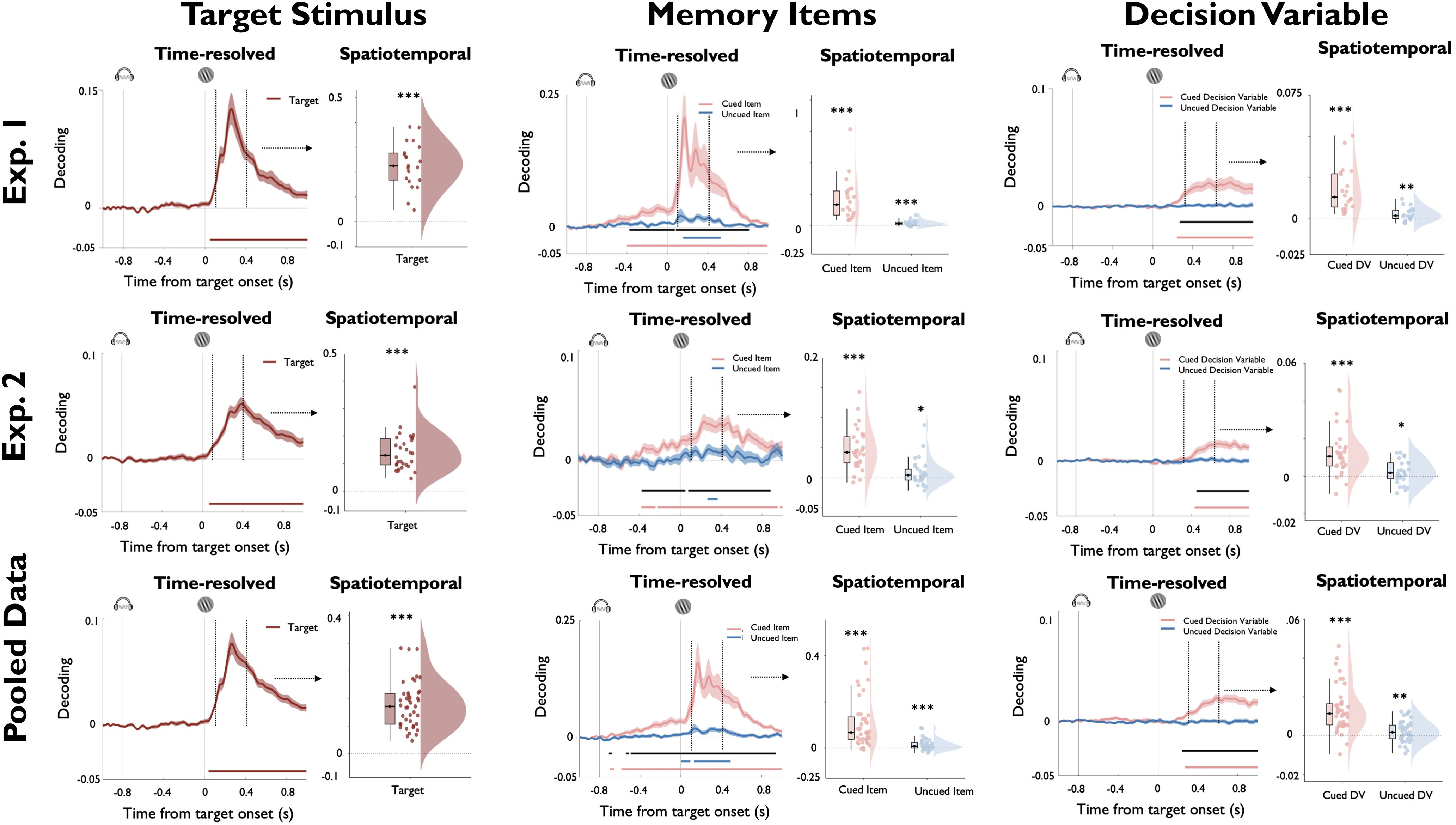
Time-resolved and spatiotemporal decoding of task variables for experiment 1 (first row), experiment 2 (second row), and the pooled data across experiments (third row). Panels on the left display the decoding results for the orientation of the target stimulus, middle panels display the decoding results for the orientation for the cued and uncued memory items, and panels on the right display the decoding results for the absolute value of the decision variable, calculated separately with respect to the cued and uncued memory item. For each experiment and task variable, plots on the left display time-resolved decoding results from 200 ms before cue onset until 800 ms after target onset. Colored lines represent cluster-corrected time periods within which decoding was significantly greater than chance. Black lines in the middle and bottom rows indicate cluster-corrected time periods within which decoding strength of the cued and uncued WM items differed significantly. Shading indicates SEM. Plots on the right display results of the spatiotemporal decoding approach where data entered into the decoder were pooled within a time window from 100 to 400 ms following target onset (for details, see text). Boxplots, Center lines indicate the median, the box outlines indicate the 25th and 75th percentiles, and the whiskers indicate 1.5 times the interquartile range. ***p < 0.005; **p < 0.01; *p < 0.05.

**Figure 4.**
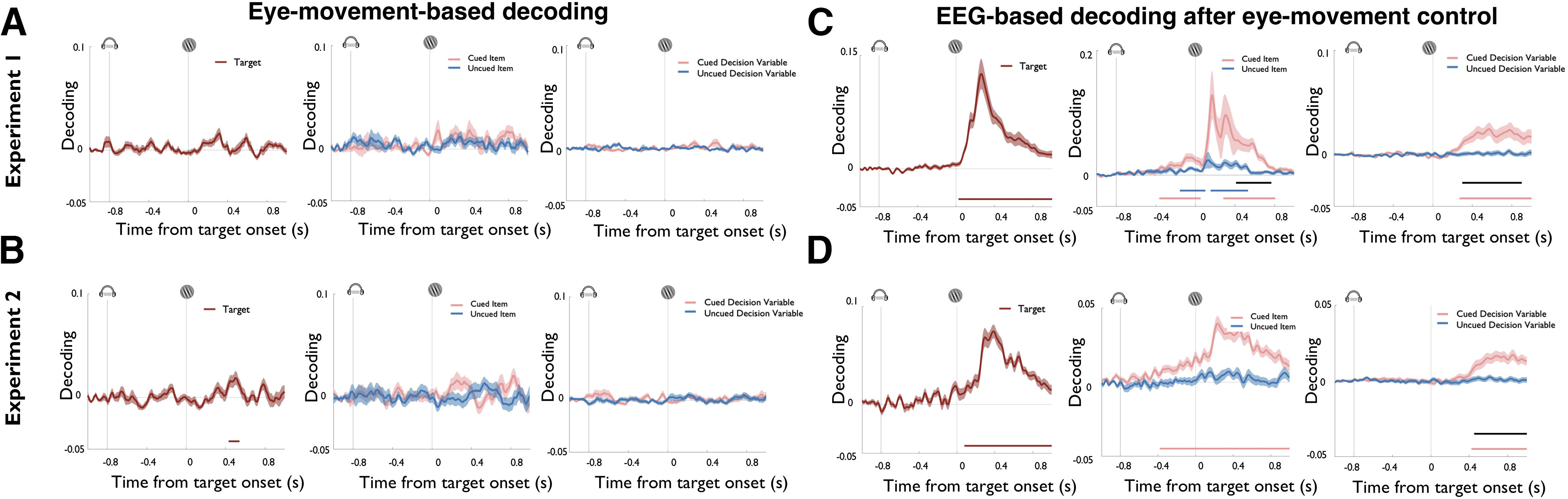
***A***, ***B***, Time-resolved decoding of task variables using EOG channels in experiment 1 (***A***, top row) and the high-resolution eye-tracker in experiment 2 (***B***). These data show that eye channels did not contain reliable information about the task variables that were decoded from EEG sensor patterns. Each plot displays time-resolved decoding results from 200 ms before cue onset until 800 ms after target onset. Colored lines represent cluster-corrected time periods within which decoding was significantly greater than chance. Shading indicates the SEM. ***C***, ***D***, To rule out that the signals decoded from EEG activity were not caused by subtle eye movements, we also regressed decoding time series obtained with the eye channels against the respective time series obtained with the EEG. Plots indicate the residual EEG signal after regressing out variance that could be explained by eye-channel signals in experiment 1 (***A***) and experiment 2 (***B***). No qualitative changes were observed compared with the original EEG decoding analysis, corroborating that eye movements did not explain the observed decoding results.

Information about the orientation of the target stimulus started to be encoded in EEG sensor patterns briefly after target onset, and decoding remained significant for the remainder of the selected time window (Fig. 3; experiment 1: from 72 ms after the target; cluster-corrected *p* < 0.0001; experiment 2: from 68 ms after the target; cluster-corrected *p* < 0.0001). Cued and uncued memory items were both decodable, but with clear differences in their respective time courses. Decoding of the cued item started to rise during delay period and was further magnified after target onset (experiment 1: first cluster: 208–376 ms after the cue; cluster-corrected *p* = 0.011; second cluster = 400–2000 ms after the cue; cluster-corrected *p* < 0.00,001; experiment 2: 420–2000 ms after the cue; cluster-corrected *p* < 0.0001). In contrast, the uncued item was not decodable during the delay period, but there was a time window of significant decoding after the onset of the target stimulus (experiment 1: 192–528 ms after the target; cluster-corrected *p* = 0.0005; experiment 2: 272–316 ms after the target; cluster-corrected *p* = 0.048). Slightly later during the target period, we could decode a decision variable for the cued item (experiment 1: 244–1000 ms after the target; cluster-corrected *p* = 0.0002; experiment 2: 388–1000 ms after the target; cluster-corrected *p* = 0.001), whereas no decision variable was decodable for the uncued item. Finally, for all three task variables, we also validated that eye movements could not explain the pattern of EEG decoding results (Fig. 4).

### Spatiotemporal decoding

In a second step, we used a complementary analysis approach to decode the same task variables from longer time windows of stimulus-evoked brain activity (Fig. 1; see Materials and Methods). Previous work has shown that such pooling over adjacent time points within trials can increase decoding sensitivity at the expense of temporal precision (Wolff et al., 2020a,b). In keeping with these studies, we pooled data points over a time period from 100 to 400 ms after target onset and treated individual trials as discrete events. As seen in Figure 3, this approach clearly enhanced decoding sensitivity. Specifically, we could reliably decode the orientation of the target (experiment 1: *t*_(19)_ = 11.606; *p* < 0.001; *d* = 2.533; experiment 2: *t*_(29)_ = 11.465; *p* < 0.001; *d* = 2.093), the orientation of the cued item (experiment 1: *t*_(19)_ = 5.443; *p* < 0.001; *d* = 1.217; experiment 2: *t*_(29)_ = 7.631; *p* < 0.001; *d* = 1.393), and the orientation of the uncued item (experiment 1: *t*_(19)_ = 4.237; *p* < 0.001; *d* = 0.947; experiment 2: *t*_(29)_ = 2.122; *p* = 0.021; *d* = 0.387). Interestingly, using the spatiotemporal approach, we could not only decode a decision variable for the cued item (experiment 1: *t*_(19)_ = 5.632; *p* < 0.001; *d* = 1.259; experiment 2: *t*_(29)_ = 5.482; *p* < 0.001; *d* = 1.001), but also for the uncued item (experiment 1: *t*_(19)_ = 2.712; *p* = 0.008; *d* = 0.606,; experiment 2: *t*_(29)_ = 2.151; *p* = 0.020; *d* = 0.393).

#### 

##### Regression analyses

#### Active, but not latent, WM states predict trial-wise variance in task performance

**Figure 5.**
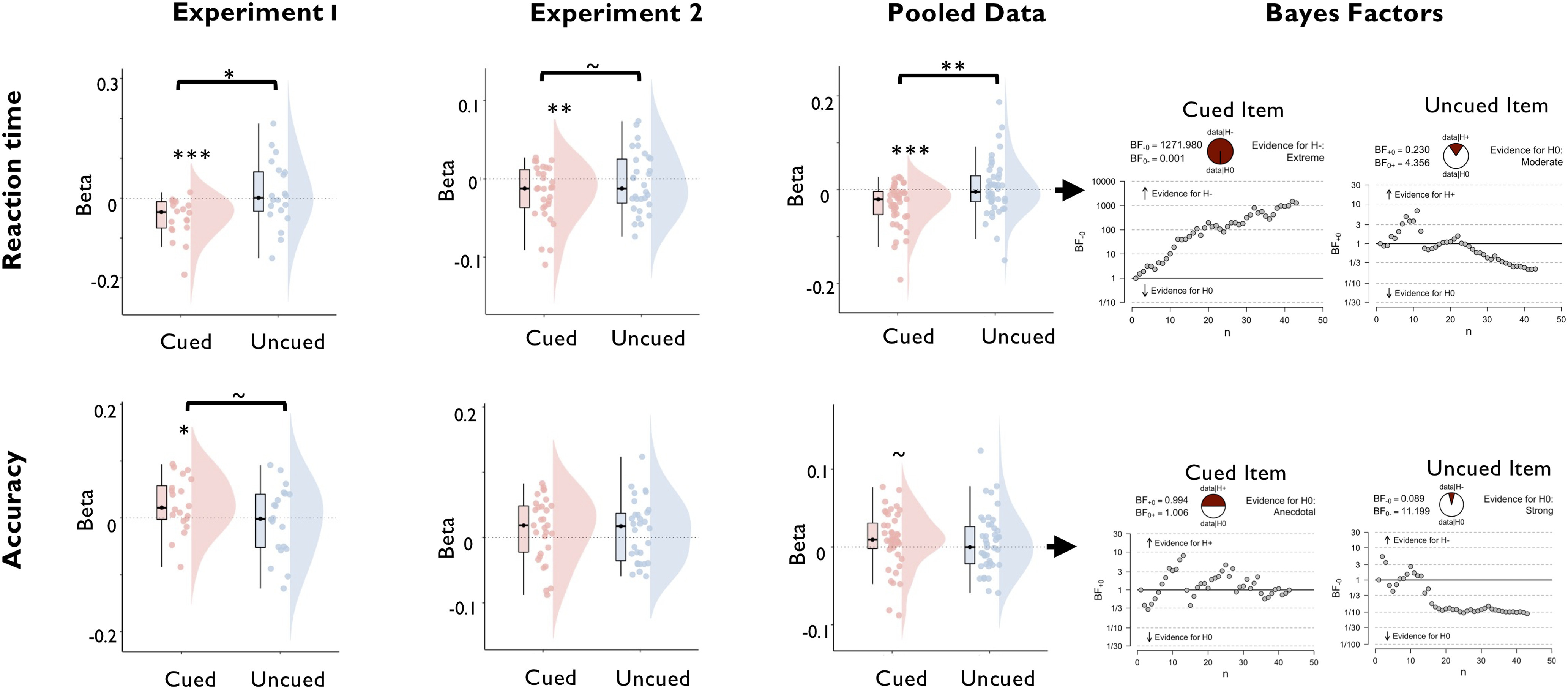
Regression of task performance based on trial-wise variance in decoding strength of the cued and uncued WM items, separately for experiment 1 and experiment 2, and the pooled data across experiments. The top row displays the distribution of regression weights across participants for the prediction of reaction time, and the bottom row displays the prediction of accuracy. Boxplots, Center lines indicate the median, the box outlines indicate the 25th and 75th percentiles, and the whiskers indicate 1.5 times the interquartile range. ***p < 0.005; **p < 0.01; *p < 0.05. Plots on the right display sequential Bayes factors for the respective regression weights (based on the pooled dataset).

We next conducted a series of regression analyses to establish the relevance of the decoded memory signals for task performance and test our hypothesis that cued and uncued items are encoded in qualitatively different functional states. To reiterate, we expected that the cued item would be encoded in a decision-oriented state that directly predicts the variance in performance, whereas the uncued item should be held in a functionally latent state that has minimal impact on current performance. To test this prediction, we regressed log-RT and accuracy against the trial-wise decoding strength of cued and uncued WM items using the spatiotemporal classifier (see Materials and Methods). As shown in Figure 5, we observed that higher trial-wise decoding of the cued item reliably predicted faster log-RT [experiment 1: *t*_(19)_ = −4.362; *p* < 0.001; *d* = −0.975; BF in favor of the alternative hypothesis (BF_H1_) = 190.357; experiment 2: *t*_(29)_ = −2.895; *p* = 0.004; *d* = 0.529; BF_H1_ = 11.950; pooled data: *t*_(42)_ = −4.637; *p* < 0.001; *d* = −0.707; BF_H1_ = 1271.980] and tended to predict higher accuracy (experiment 1: *t*_(19)_ = 2.207; *p* = 0.020; *d* = 0.493; BF_H1_ = 3.239; experiment 2: *t*_(29)_ = 1.504; *p* = 0.072; *d* = 0.275; BF_H1_ = 0.985; pooled data: *t*_(42)_ = 1.596; *p* = 0.059; *d* = 0.243; BF_H1_ = 0.994). By contrast, trial-wise variance in decoding of the uncued item predicted neither log-RT [experiment 1: *t*_(19)_ = 0.622; *p* = 0.271; *d* = 0.139; BF in favor of the null hypothesis (BF_H0_) = 2.515; experiment 2: *t*_(29)_ = −0.713; *p* = 0.759; *d* = −0.130; BF_H0_ = 8.172; pooled data: *t*_(42)_ = 0.391; *p* = 0.349; *d* = 0.060; BF_H0_ = 4.356] nor accuracy (experiment 1: *t*_(19)_ = −0.410; *p* = 0.343; *d* = −*0*.092; BF_H0_ = 3.072; experiment 2: *t*_(29)_ = 1.186; *p* = 0.877; *d* = 0.216; BF_H0_ = 10.324; pooled data: *t*_(42)_ = 0.986; *p* = 0.835; *d* = 0.150; BF_H0_ = 11.199), with Bayes factors indicating substantial evidence in favor of the respective null hypotheses. Regression weights of the cued and the uncued items differed significantly in the prediction of log-RT (experiment 1: *t*_(19)_ = −2.452; *p* = 0.012; *d* = −0.548; BF_H1_ = 4.903; experiment 2: *t*_(29)_ = −1.360; *p* = 0.092; *d* = −0.248; BF_H1_ = 0.805; pooled data: *t*_(42)_ = −2.669; *p* = 0.005; *d* = −0.407; BF_H1_ = 7.413). Together, these results support our prediction that cued items are encoded in a functionally active state that guides WM-based decisions, whereas uncued items are held in a functionally latent state that has minimal impact on ongoing cognition.

#### Functionally active and latent WM states both track memory precision over longer timescales

After establishing that the decoding strength of uncued items is unrelated to performance on the current trial, we next tested the extent to which these signals nonetheless capture behaviorally relevant variance by examining their contribution to general maintenance. Although a strong representation of the uncued item is not advantageous on the current trial, it is nonetheless crucial for performance on other trials in the same block when priorities differ, and the same item becomes task relevant. Therefore, maintaining an accurate representation of the uncued memory item reflects a minimal requirement for solving the task as a whole. Accordingly, we reasoned that the overall quality with which a memory item is encoded throughout the block when it is uncued may track performance in the same block on those trials when the same item is cued. We tested this assumption by regressing log-RT and accuracy against the decoding strength of the two memory items averaged over the remaining trials of the block. Assuming that block-wise decoding tracks differences in the general quality with which items are encoded throughout the block, we reasoned that the strength of both cued and uncued items should scale positively with performance.

**Figure 6.**
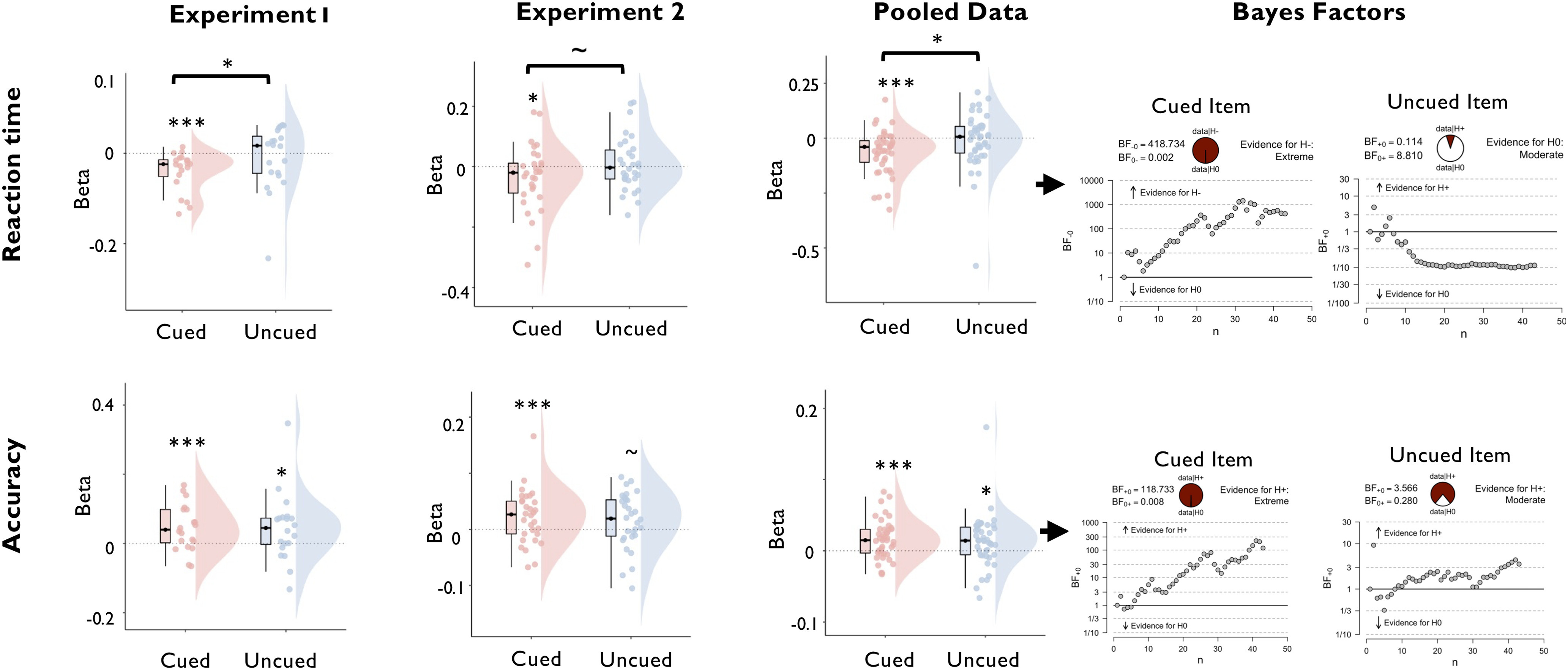
Regression of task performance based on block-wise decoding accuracy of the cued and uncued WM items, separately for experiment 1, experiment 2, and the pooled data across experiments. The top row displays the distribution of regression weights across participants for the prediction of reaction time, and the bottom row displays the same for the prediction of accuracy. Boxplots, Center lines indicate the median, the box outlines indicate the 25th and 75th percentiles, and the whiskers indicate 1.5 times the interquartile range. ***p < 0.005; **p < 0.01; *p < 0.05. Plots on the right display sequential Bayes factors for the respective regression weights (based on the pooled dataset).

As shown in Figure 6, accuracy was indeed predicted by the block-wise decoding strength of the cued item (experiment 1: *t*_(19)_ = 3.034; *p* < 0.003; *d* = 0.678; BF_H1_ = 14.020; experiment 2: *t*_(29)_ = 2.526; *p* = 0.009; *d* =0.461; BF_H1_ = 5.633; pooled data: *t*_(42)_ = 3.797; *p* < 0.001; *d* = 0.579; BF_H1_ = 177.778) and the uncued item (experiment 1: *t*_(19)_ = 2.005; *p* < 0.030; *d* = 0.448; BF_H1_ = 2.341; experiment 2: *t*_(29)_ = 1.656; *p* = 0.054; *d* = 0.302; BF_H1_ = 1.233; pooled data: *t*_(42)_ = −2.318; *p* < 0.013; *d* = 0.354; BF_H1_ = 5.725). Differences between the regression weights for the cued and the uncued items were nonsignificant, with Bayes factors providing evidence in favor of the null hypothesis (experiment 1: *t*_(19)_ = 0.045; *p* = 0.964; *d* = 0.010; BF_H0_ = 4.300; experiment 2: *t*_(29)_ = 0.826; *p* = 0.415; *d* = 0.151; BF_H0_ = 3.760; pooled data: *t*_(42)_ = 0.514; *p* = 0.695; *d* = 0.078; BF_H0_ = 5.796). In addition, block-wise decoding of the cued item also predicted log-RT (experiment 1: *t*_(19)_ = −4.093; *p* < 0.001; *d* = −0.915; BF_H1_ = 111.097; experiment 2: *t*_(29)_ = −1.812; *p* = 0.040; *d* = −0.331; BF_H1_ = 1.575; pooled data: *t*_(42)_ = −4.253; *p* < 0.001; *d* = −0.648; BF_H1_ = 155.046), which was not the case for the uncued item (all *p* > 0.299; all *d* < 0.140; all BF_H0_ > 3.253). Together, these results show that although functionally latent WM states do not engage with decision dynamics on the current trial, they nonetheless support performance at longer timescales.

#### Similarity to the functionally active state predicts interference from uncued items

The previous sections established that uncued items are encoded in a format that supports memory maintenance but has minimal impact on ongoing processing. Nonetheless, we had observed the following two behavioral signatures of interference between the two memory items: a subtle but reliable effect of the angular distance between the uncued item and the target stimulus on decision accuracy; and a performance cost on trials demanding a priority shift between items. Given our previous finding that the strength of functionally active, but not functionally latent, WM states tracks fluctuations in performance, we reasoned that interference may reflect the extent to which uncued memory items are encoded in patterns resembling their functionally active state. To test this idea, we repeated the trial-wise regression analyses used above with a cross-item decoder that was trained on data sorted by the cued item and tested on data sorted by the uncued item.

**Figure 7.**
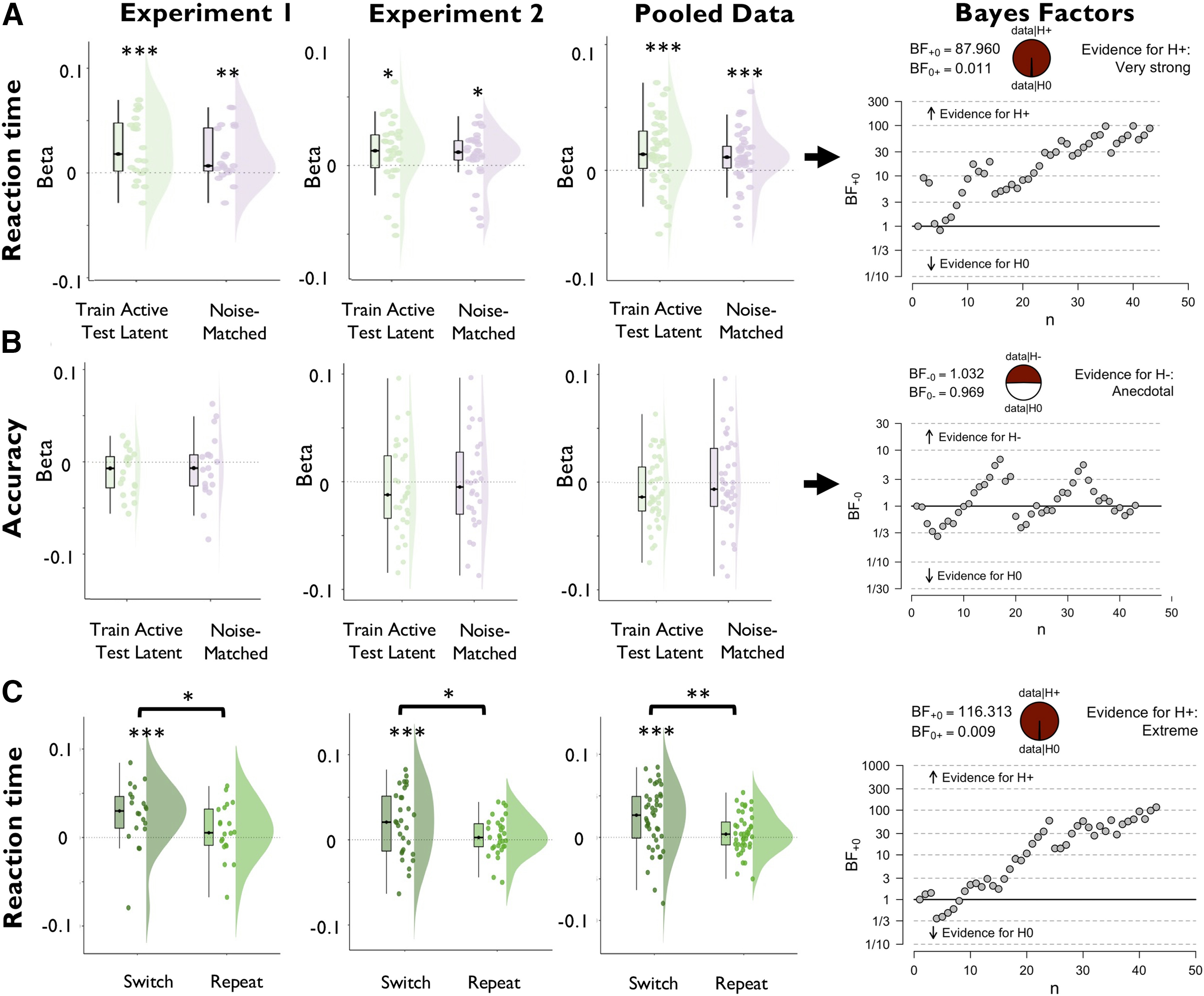
Regression of task performance based on trial-wise variance in the decoding strength of a cross-item decoder that was trained on the data sorted by the cued WM item and tested on the data sorted by the uncued WM item. Results are shown separately for experiment 1, experiment 2, and the pooled data across experiments. ***A***, ***B***, The cross-item decoder reliably predicted slower reaction time (***A***, green plots), but was unrelated to accuracy (***B***, green plots). Notably, the RT effect remained significant after adding noise to cross-item decoder output to match it in signal strength with the regular decoder of the uncued item by adding noise to the decoder output (purple plots; see Materials and Methods). ***C***, Reaction time effects of the cross-item decoder were observed only on trials that required a priority shift between WM items relative to the previous trial (switch trials), but not on trials in which the item priority was repeated (repetition trials). Plots display the distribution of regression weights across participants. Boxplots, Center lines indicate the median, the box outlines indicate the 25th and 75th percentiles, and the whiskers indicate 1.5 times the interquartile range. Plots on the right display sequential Bayes factors for the respective regression weights (based on the pooled dataset of the cross-item decoder). ***p < 0.005, **p < 0.01, *p < 0.05.

The cross-item decoder successfully classified uncued items with above-chance accuracy (experiment 1: *t*_(19)_ = 4.719; *p* < 0.001; *d* = 1.055; experiment 2: *t*_(29)_ = 2.751; *p* = 0.010; *d* = 0.502). Decoding magnitude was enhanced in comparison to the regular decoder of the uncued item in experiment 1 (*t*_(19)_ = 2.821; *p* = 0.011; *d* = 0.631), but not in experiment 2 (*t*_(29)_ = 0.605; *p* = 0.550; *d* = 0.110). Critically, higher trial-wise cross-item decoding indeed predicted slower log-RT (Fig. 7; experiment 1: *t*_(19)_ = 3.497; *p* = 0.001; *d* = 0.782; BF_H1_ = 34.074; experiment 2: *t*_(29)_ = 1.996; *p* = 0.028; *d* = 0.364; BF_H1_ = 2.135; pooled data: *t*_(42)._= 3.685; *p* < 0.001; *d* = 0.562; BF_H1_ = 87.960) and tended to predict reduced accuracy (experiment 1: *t*_(19)_ = −1.980; *p* = 0.031; *d* = −0.443; BF_H1_ = 2.250; experiment 2: *t*_(29)_ = −0.772; *p* = 0.223; *d* = 0.102; BF_H0_ = 2.545; pooled data: *t*_(42)._ = 1.619; *p* = 0.056; *d* = −0.247; BF_H1_ = 1.032). Notably, the regression weights for log-RT were significantly larger than those of the regular decoder of the uncued item (experiment 1: *t*_(19)_ = 3.037; *p* = 0.003; *d* = 0.679; BF_H1_ = 14.102; experiment 2: *t*_(29)_ = 1.907; *p* = 0.033; *d* = 0.348; BF_H1_ = 1.837; pooled data: *t*_(42)_ = 3.201; *p* = 0.001; *d* = 0.488; BF_H1_ = 25.515). We tested whether the difference between the two decoders could be attributed to differences in signal strength by adding noise to the cross-item decoder to match it with the regular decoder in terms of mean and SD (Materials and Methods). Importantly, the interference effects with log-RT remained significant after noise matching (experiment 1: *t*_(19)_ = −2.818; *p* = 0.011; *d* = 0.630; BF_H1_ = 4.741; experiment 2: *t*_(29)_ = −1.889; *p* = 0.034; *d* = 0.345; BF_H1_ = 1.786; pooled data: *t*_(42)_ = −3.158; *p* = 0.001; *d* = 0.482; BF_H1_ = 22.922), emphasizing that differences in signal strength do not account for differences between functional states (Fig. 7*A*,*B*).

We reasoned that instances of cross-item interference might be expressed most strongly on trials demanding priority shifts between items, and therefore repeated the foregoing analysis separately for trials incurring priority shifts, relative to the previous trial (switch trials), and trials that did not (repetition trials). Indeed, the negative prediction of log-RT was significant only on switch trials (experiment 1: *t*_(19)_ = 3.259; *p* = 0.002; *d* = 0.729; BF_H1_ = 21.499; experiment 2: *t*_(29)_ = 2.774; *p* = 0.005; *d* = 0.506; BF_H1_ = 9.270; pooled data: *t*_(42)_ = 3.790; *p* < 0.001; *d* = 0.578; BF_H1_ = 116.313), but not on repetition trials (experiment 1: *t*_(19)_ = 1.127; *p* = 0.137; *d* = 0.252; BF_H0_ = 1.450; experiment 2: *t*_(29)_ = 0.961; *p* = 0.172; *d* = 0.175; BF_H0_ = 2.060; pooled data: *t*_(42)_ = 1.347; *p* = 0.093; *d* = 0.205; BF_H0_ = 1.452), and the difference between trial types was significant (experiment 1: *t*_(19)_ = 2.034; *p* = 0.028; *d* = 0.455; BF = 2.450; experiment 2: *t*_(29)_ = 1.966; *p* = 0.029; *d* = 0.359; BF = 2.028; pooled data: *t*_(42)_ = 2.675; *p* = 0.005; *d* = 0.408; BF_H1_ = 7.513). Collectively, these results show that interference between WM items arises because of lingering neural patterns coding for previously acted upon, but no longer immediately task-relevant, items.

### Drift diffusion modeling

**Figure 8.**
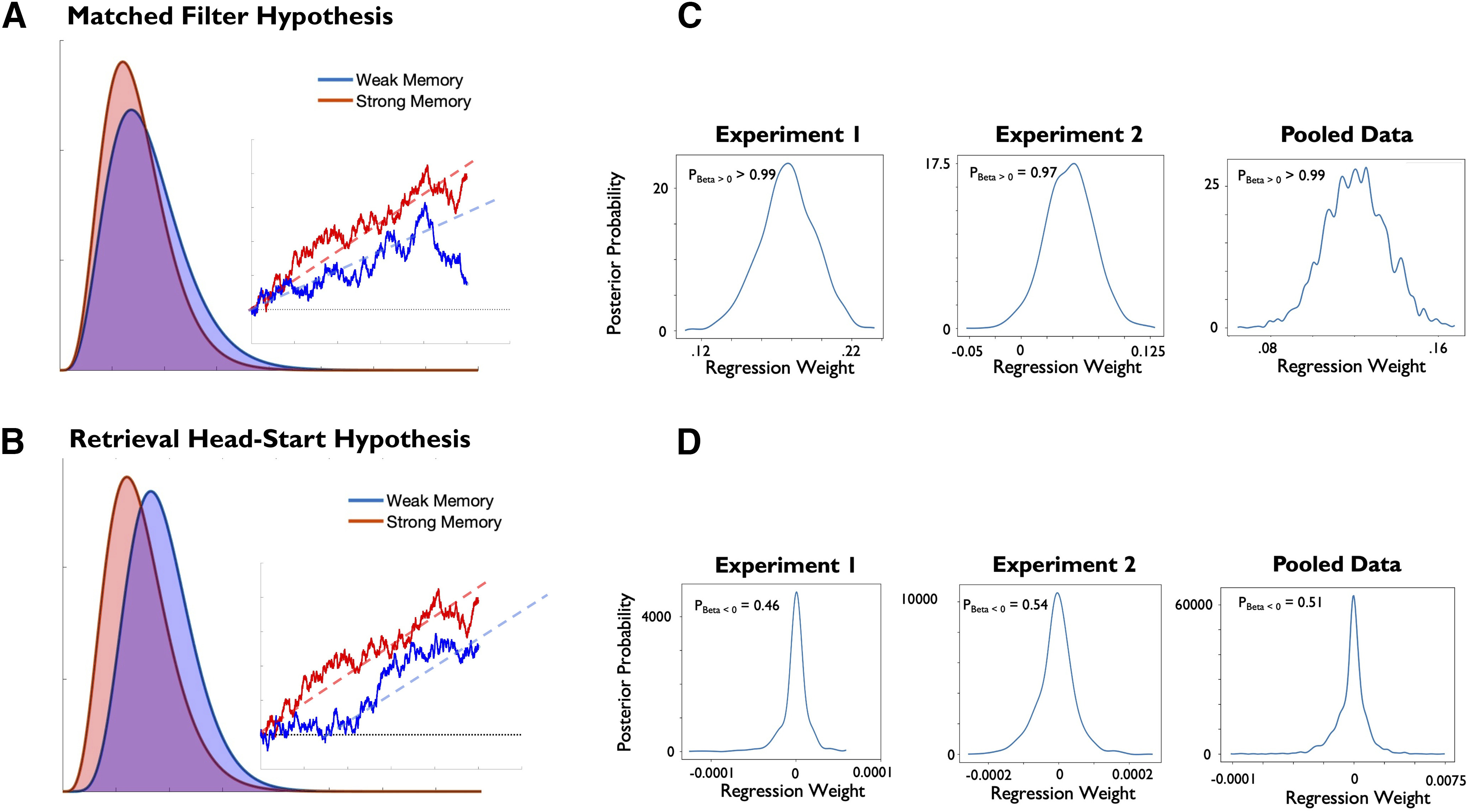
Drift diffusion modeling of task performance as a function of decoding strength of the active WM item. ***A***, ***B***, Illustration of the two hypotheses under investigation that predict that trial-wise variance in decoding of the cued item tracks either changes in drift rate (matched filter hypothesis) or non-decision time (retrieval head-start hypothesis). The plots display simulated RT distributions and exemplary single-trial diffusion patterns under the two accounts. ***C***, ***D***, Results of the DDM regression analysis that predicted trial-wise changes in drift rate (***C***) and non-decision time (***D***) based on trial-wise changes in decoding strength of the cued item. Plots display the posterior distribution of estimated regression weights, resulting from 5000 iterations from which the initial 1000 iterations were discarded as burn-in. The significance of effects was evaluated by quantifying the amount of the posterior probability mass that was in the predicted direction (positive for drift rate and negative for non-decision time; for details, see Materials and Methods, and Results). As indicated on the plots, decoding of the cued item reliably predicted changes in drift rate, but it was not associated with changes in non-decision time.

We conducted another set of analyses to further characterize the format of functionally active WM states and obtain more detailed insights into the mechanisms by which they influenced decisions in our task by comparing two different explanations for the link between decoding strength and performance (Fig. 8, illustration). First, the strength of the functionally active state may determine the ease with which sensory input is interpreted. This would be the case if the functionally active WM item acts as a matched filter that directly feeds in the accumulation of decision-related evidence (Hayden and Gallant, 2013; matched filter hypothesis). Second, the strength of the functionally active state may determine the ease with which the item can be retrieved for decision-making (Souza et al., 2016); thus, providing a head start for decisions on trials with a strong representation of the cued item (retrieval head-start hypothesis).

To evaluate these possibilities, we fit a set of DDMs to our behavioral data and related variance in model parameters to variance in decoding. We focused on the following two parameters of the DDM: drift rate and non-decision time. The drift rate reflects the quality with which decision-relevant information is integrated, and scales negatively with categorization difficulty. The non-decision time reflects the time needed for processes that are not directly related to evidence accumulation such as the encoding of a stimulus or the execution of a response. To test the accounts outlined above, we fit a set of linear regression models predicting trial-wise changes in the two decision parameters based on trial-wise changes in decoding strength of the cued memory item (see Materials and Methods). The matched filter hypothesis predicts that stronger decoding is associated with higher drift rates, whereas the retrieval head-start hypothesis predicts that stronger decoding is associated with reduced non-decision time (Fig. 8*A*,*B*). In support of the matched filter hypothesis, we observed reliable positive regression weights for predictions of drift rate in both experiments (experiment 1: *p*_Beta > 0_ = 0.99; experiment 2: *p*_Beta > 0_ = 0.97; pooled data: *p*_Beta > 0_ = 0.99), whereas regression weights for the non-decision time were indistinguishable from 0 (experiment 1: *p*_Beta < 0_ = 0.46; experiment 2: *p*_Beta < 0_ = 0.54, pooled data: *p*_Beta < 0_ = 0.51).

### Priority shifts and WM precision

**Figure 9.**
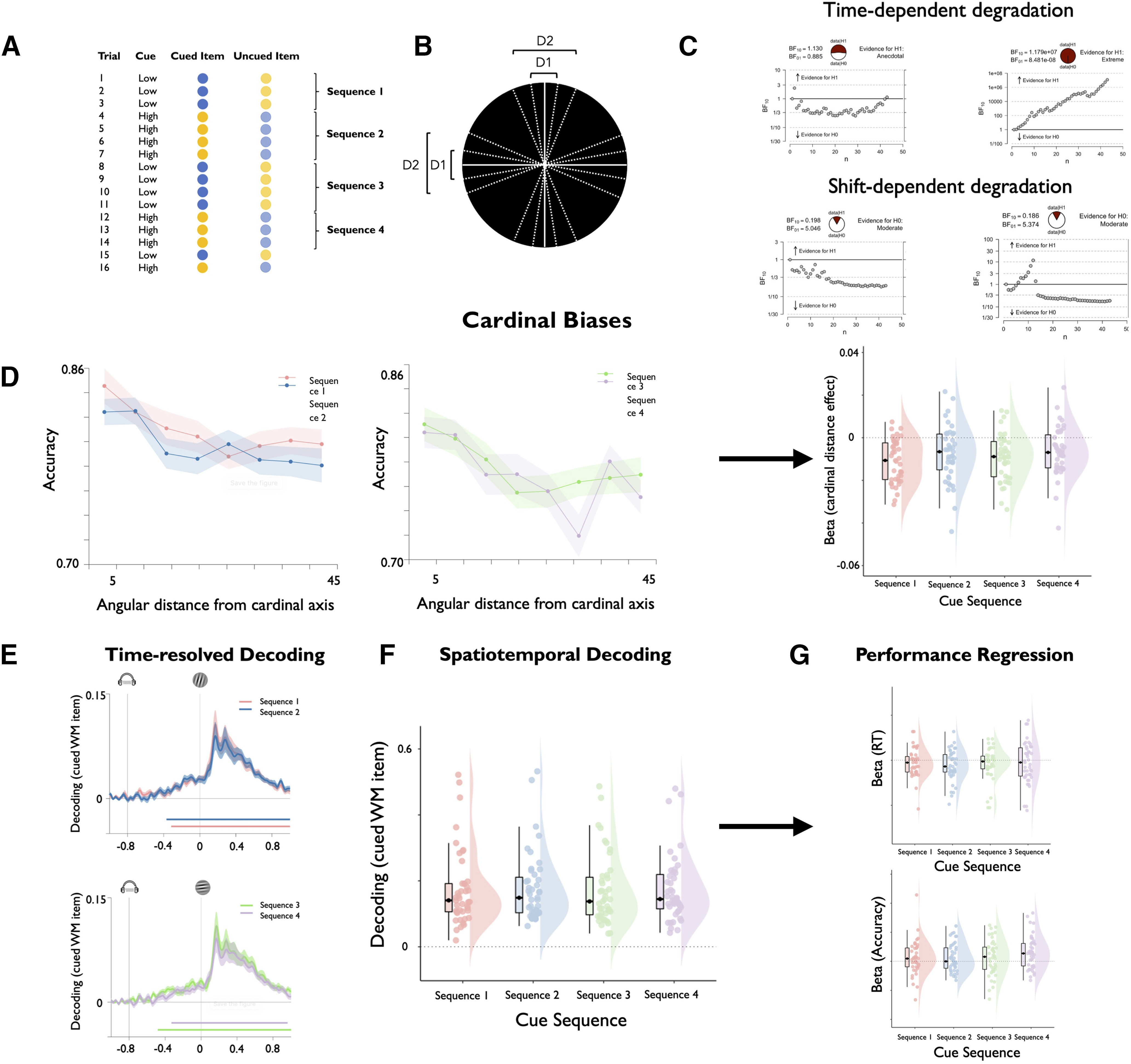
Representational quality of WM items as a function of priority shifts. ***A***, Illustration of a hypothetical trial sequence within a task block and the resulting cue sequences that describe periods within the block, wherein an item was used before and after a priority shift. ***B***, Illustration of cardinal distances of WM items. For each WM item, we calculated the angular distance to the closest cardinal axis (vertical or horizontal). Note that this visualization displays a simplified case with only two different distances, whereas our stimulus set contained a total of eight different cardinal distances. ***C***, Performance was regressed against the trial number within the current block and the cue sequence within the current block to index time-dependent and shift-dependent degradation of WM items, respectively. The plots display sequential Bayes factors of the respective regression weights for log-RT (left) and accuracy (right). Our results revealed robust effects of time-dependent degradation, but no effects for shift-dependent degradation with Bayes factors lending support for the null hypotheses. ***D***, Performance accuracy was also regressed against the angular distance between the cued WM item and the closest cardinal axis (see ***B***), separately for each cue sequence. The left and the middle plot display mean accuracy for the respective conditions, and the right plot displays the regression weights for each cue sequence. As shown on the plots, we observed robust cardinal distance effects, whereby accuracy decreased with larger distances, but these cardinal bias effects did not differ between cue sequences. ***E–G***, We also compared decoding time courses (***E***), spatiotemporal decoding strength (***F***), and the strength of performance regression (***G***) of cued WM items among the first four cue sequences. No significant differences were observed. All of these results converge on the notion that priority shifts did not degrade the quality of WM representations. Please note that results are shown for the pooled data across both experiments.

In the final analyses, we aimed to further characterize the format of functionally latent WM states by testing whether shifting priority away from an item would perturb its precision. As outlined above, several WM theories assume that uncued items are degraded versions of cued items (e.g., because they may receive a smaller part of a limited cognitive resource for WM maintenance; Zhang and Luck, 2008; Ma et al., 2014). In contrast, state-based theories assume that cued and uncued items differ only with regard to their functional properties but not with regard to their precision (Oberauer, 2009; Myers et al., 2017). Importantly, the block structure of our task, with multiple sequences of item priority (i.e., cue sequences; Fig. 9*A*), provided a unique window to adjudicate between these models by examining the precision of WM items before and after priority shifts. Whereas resource theories predict that shifting priority away from an item will degrade its precision, state-based theories predict that memory precision should be unaffected by priority shifts.

Initially, we used multiple regression to test whether the number of priority shifts within a block would predict declines in accuracy beyond the aforementioned time-dependent decline (see Materials and Methods). As seen in Figure 9*C*, accuracy declined only as a function of the number of trials within the block (experiment 1: *t*_(19)_ = −7.212; *p* < 0.001; *d* = −1.613; BF_H1_ = 46 386.592; experiment 2: *t*_(29)_ = 5.382; *p* < 0.001; *d* = −0.983; BF_H1_ = 4761.628; pooled data: *t*_(42)_ = −7.833; *p* < 0.001; *d* = −1.194; BF_H1_ = 1.179e*7), but not as a function of the number of priority shifts (experiment 1: *t*_(19)_ = −0.799; *p* = 0.217; *d* = −0.179; BF_H0_ = 2.100; experiment 2: *t*_(29)_ = 1.768; *p* = 0.956; *d* = 0.323; BF_H0_ = 12.961; pooled data: *t*_(42)_ = 0.505; *p* = 0.616; *d* = 0.077; BF_H0_ = 5.374).

We next examined whether priority shifts would enhance categorical biases, whereby WM items are encoded with respect to the closest cardinal axis (Bae and Luck, 2019; Fig. 9*B*; see Materials and Methods). Accuracy indeed decreased with larger cardinal distances (experiment 1: *t*_(19)_ = −5.801; *p* < 0.001; *d* = −1.297; BF_H1_ = 3307.866; experiment 2: *t*_(29)_ = −6.762; *p* < 0.001; *d* = −1.235; BF_H1_ = 156142.464; pooled data: *t*_(42)_ = 8.475; *p* < 0.001; *d* = −1.292; BF_H1_ = 8.336e*7), consistent with the presence of categorical biases. Importantly, however, the magnitude of cardinal distance effects did not differ among the first four cue sequences (Fig. 9*D*; all *p* > 0.441; all *d* < 0.165; all BF_H0_ > 3.263), corroborating that priority shifts did not degrade WM precision.

Last, we compared the EEG-based decoding strength of WM items for the first four cue sequences of each block (see Materials and Methods). Consistent with the foregoing sections, we observed no significant differences among cue sequences in terms of decoding time courses (Fig. 9*E*; all cluster-corrected *p* > 0.353; all *d* < 0.145; all BF_H0_ > 2.213), spatiotemporal decoding strength (Fig. 9*F*; all *p* > 0.144; all *d* < 0.241; all BF_H0_ > 1.929), or in the link between decoding strength and performance (Fig. 9*G*; all *p* > 0.508; all *d* < 0.124; all BF_H0_ > 2.354). Altogether, these results show that priority shifts did not degrade memory precision, providing convergent evidence for state-based theories of WM.

## Discussion

Our results show that the use of WM for guiding behavior relies on an active reconfiguration that transforms purely mnemonic states into an action-oriented format. This view aligns with neuropsychological dissociations between WM maintenance and use (Duncan et al., 1996), and with behavioral evidence that cued, but not uncued, WM items can bias perception toward memory-matching stimuli (Olivers et al., 2011). However, previous attempts to characterize WM representations functionally through neuroimaging have been hindered by the fact that neural signals coding for uncued items are typically very subtle and therefore difficult to examine. Here, we were able to recover neural traces of cued and uncued items from stimulus-evoked EEG signals, permitting us to characterize their functional contributions to WM-based behavior. Uncued items were encoded in a functionally latent state that did not engage with ongoing processing but was predictive of performance accuracy at a block-wise timescale. This highlights a contribution to decision-making by providing a storage format that protects memories from decay, while minimizing interference with currently prioritized cognition. Such a coding scheme is critical for many complex and time-extended behaviors incurring multiple nested components and priority shifts among them (Holroyd et al., 2018). Understanding how the brain structures cognition over such intermediate timescales will be key for developing more integrative theories of memory-guided behavior (Hasson et al., 2015; Kamiński, 2017), and we anticipate that such research may reveal maintenance contributions by structures in the medial temporal lobe that have classically been associated with long-term memory (Olsen et al., 2009).

In contrast to the functionally latent state, cued items were encoded in a functionally active state that predicted dynamic trial-wise changes in performance, especially reaction time. Interestingly, these effects were specifically tied to changes in drift rate, while leaving the non-decision time unaffected. This suggests that functionally active WM items act as matched filters that directly transform sensory information into task-dependent decision variables (e.g., by weighting the target-evoked patterns by mnemonic patterns coding for the cued WM item; Sugase-Miyamoto et al., 2008). Conversely, this result is at odds with proposals attributing the benefits of prioritization within WM to the facilitation of retrieval processes, which may give decisions a head start, compared with decisions without prioritization (Souza et al., 2016). Notably, the similarity of uncued items to their functionally active state also tracked the amount of interference from those items after priority shifts. This interference was transient and did not cause the degradation or forgetting of WM items on subsequent trials, consistent with proposals that the updating of WM contents reflects an active and time-consuming process (Oberauer, 2003), and that failure to complete it ahead of time leads to interference during target processing (Monsell, 2003).

Importantly, conceiving prioritization within WM as a transition from functionally latent mnemonic states toward functionally active task-oriented states provides a new perspective on other established experimental phenomena such as the effects of retrospective cuing. These effects have most commonly been explained by assuming a finite cognitive resource for WM maintenance that can be continuously distributed among different items (Ma et al., 2014). From this perspective, a more selective focus improves memory for cued items, but at the cost of degraded memory for uncued items. The account we propose offers an alternative explanation, whereby the benefits of retrospective cues reflect, at least in part, the reformatting of purely mnemonic states into a prospective action rule that enforces task-relevant stimulus–response mappings. From this perspective, capacity limitations also concern behavioral readout, in addition to basic maintenance, when assuming that only a single item can be encoded as an action rule at any given moment (Olivers et al., 2011). It also implies that selecting an item for guiding behavior should not necessarily impede the maintenance of other concurrently held items, because once the selected item is encoded in a rule-like state, it is no longer necessary to sustain selective attention to the corresponding mnemonic representation. This view of state transitions as transient events mirrors the time course of lateralized alpha band suppression during the prioritization of spatially separated WM items (de Vries et al., 2020), and could also explain previous findings that the beneficial effects of retrospective cues can sometimes prevail even when attention is diverted from the cued item (Rerko et al., 2014) or that they can occur in the absence of reliable performance costs for uncued items (Gunseli et al., 2015; Myers et al., 2018).

A central question for future research will be to delineate the precise neurophysiological mechanisms that underpin functional WM states. One possibility is that functionally active and latent WM states directly map onto corresponding activity states: neurally active and “activity-silent” (Manohar et al., 2019; Kamiński and Rutishauser, 2020). Our results are consistent with this view, as spontaneous delay activity encoded only the cued item, but we identified traces of both items in the target-evoked EEG signal, consistent with an impulse response uncovering hidden neural states (Wolff et al., 2017). However, we note that our current study was not designed to test this specific question but instead focused on dissociating WM states functionally. As we have discussed in detail previously, it is theoretically possible for activity states, or activity-silent states, to support functionally active or latent cognitive states (Stokes et al., 2020). The only formal requirement is that their representational format differs qualitatively. This could be achieved through a division of labor between activity-based and activity-silent mechanisms, but also through qualitative differences in the exact patterns (van Loon et al., 2018), or brain areas (Christophel et al., 2018), for the respective functional states. In any case, we assert that future research should shift from focusing merely on the presence or absence of decodable memory signals toward characterizing their functional properties and the mechanisms that permit their transformation.

One such topic for future inquiry will be to compare the functional states we identified with different mnemonic coding schemes, especially the possibility that cued and uncued items could be represented in inverted activation patterns in the same cortical regions. Two recent fMRI studies manipulated priority states within individual trials and observed negative cross-decoding between cued and uncued items (van Loon et al., 2018; Yu et al., 2020), suggestive of opponent coding. At first blush, our findings are inconsistent with this proposal, as we observed positive rather than negative cross-item decoding in our task. There are numerous differences between the two studies and ours, so we can only speculate about the source of this discrepancy. For example, the different brain recording methods may have captured different aspects of the underlying neural signals with EEG being more sensitive to rapid stimulus-evoked signals and fMRI being more sensitive to tonic and regionally specific signals. It is also possible that sensory impulses, such as our target stimuli, change the similarity between patterns coding for cued and uncued WM items. Notably, inverted coding has also been observed in target-evoked brain signals (van Loon et al., 2018); so, this factor alone is unlikely to account for the differences between studies. Nonetheless, it will be incumbent for the field to develop methods capable of measuring spontaneous neural states coding for uncued WM items in the absence of sensory stimulation. We generally assume that independent coding schemes for cued and uncued items confer general advantages over inverted schemes, as they permit robust maintenance of both items while minimizing mutual interference. However, task demands may alter the optimality of different formats. For instance, inverted representations might become advantageous when simultaneously maintained items are encoded in nonoverlapping neural populations, when response contingencies are not explicit before probe onset, or when readout demands require maximal disambiguation between memory items (Geng et al., 2017). Therefore, more research will be necessary to establish the boundary conditions of different mnemonic coding schemes.

In conclusion, our results delineate a hierarchical model of WM wherein a single item is stored in a qualitatively different format to concurrently held items. The prioritized format is functionally active and implements a task-relevant transformation of sensory input into decision evidence, whereas other items are stored in a functionally latent format that does not interact with ongoing processing. Importantly, prioritization is highly flexible and dynamic, whereby latent states form the basic neural substrate for maintenance and can be used to implement the active representation when needed.
